# Effect of COVID-19 on Pregnancy and Neonate's Vital Parameters: A Systematic Review

**DOI:** 10.1155/2023/3015072

**Published:** 2023-05-13

**Authors:** Anna Charuta, Monika Smuniewska, Zofia Woźniak, Agnieszka Paziewska

**Affiliations:** ^1^Siedlce University of Natural Sciences and Humanities, Institute of Health, Faculty of Medical and Health Sciences, Poland; ^2^Siedlce University of Natural Sciences and Humanities, Institute of Health, Faculty of Medical and Health Sciences, Mazowiecki Provincial Hospital in Siedlce Named after Saint John Paul II in Siedlce, Poland; ^3^Siedlce University of Natural Sciences and Humanities, Institute of Health, Faculty of Medical and Health Sciences, Independent Public Health Care Center in Sokołów Podlaski, Poland

## Abstract

**Background:**

COVID-19 is a new pandemic, which was declared by the World Health Organization in 2019 as a threat to public health. According to numerous reports, it can have negative consequences for pregnant women, labour, and neonates born to infected mothers. The aim of this paper was to gather the evidence and to present a summary of the results of studies concerning COVID-19 in pregnant women and their neonates.

**Methods:**

Articles from prestigious journals covering the period from 2020 to February 2023, relevant review papers, and original research articles from PubMed were analysed. In order to analyse the available research literature, the Web of Science, Scopus, and PubMed databases were used, in which the search for articles was conducted using terms (“pregnancy,” “coronavirus,” “SARS-CoV-2,” and “newborn”) and using PRISMA (Preferred Reporting Items for Systemic Reviews and Meta-Analysis) guidelines for clinical trials. Meta-analyses and systematic reviews (2022–2023) on symptoms, neonatal course, and risk of COVID-19 infection have been summarized. Summary of meta-analyses and systematic reviews (2022–2023) on the effect and adverse reaction of the COVID-19 vaccination is presented.

**Results:**

As a result of the research conducted, it was confirmed that in most pregnant women, no serious signs of the infection were observed, although isolated cases of death related to COVID-19 in pregnant women were reported. Several authors called attention to the more severe course of the infection in pregnant women with obesity. It seemed that no vertical transmission from mother to child was occurring. Nevertheless, the information was not clinching. The condition of the neonates born to mothers with COVID-19 was in most cases described as normal; however, some papers reported deaths of infected neonates.

**Conclusions:**

Due to insufficient data, further research is necessary. Further studies and follow-up are recommended, which would make possible an assessment of remote effects of COVID-19 on pregnancy and vital parameters of the newborn.

## 1. Background

Pregnancy is a special time in a woman's life. Each woman planning pregnancy tries to prepare her body for that exceptional, but also observation- and care-requiring, period.

The preparation of the mother's body for pregnancy should be started about 12 months before the planned pregnancy [1]. Wade et al. [2] suggest that a control gynaecological examination would direct the care of pregnant women to avoid failures in the early pregnancy period.

Anatomical and physiological changes during pregnancy impact the respiratory (shape of the chest and elevation of the diaphragm), immune, and cardiovascular systems. Additionally, changes in metabolism are observed.

The diaphragm position during pregnancy, resulting from displacement of the internal organs, reduces the tolerance of hypoxia [3]. Dashraath et al. [4] call attention to the fact that during pregnancy, a shallowing of the physiological breathing and reduction in total lung capacity result in an increase of the oxygen requirement of the pregnant woman. Pregnancy and the developing foetus can cause dyspnoea, which should be distinguished from a pathology or disease. During pregnancy, a change of the lung volume occurs together with a dilation of the blood vessels, which can lead to an increased amount of secretion in the respiratory tract and, as a consequence, can create favourable conditions for infections.

Infections, particularly viral ones, during pregnancy frequently result in serious consequences for the developing foetus [5, 6]. The appearance in 2019 of a new coronavirus called SARS-CoV-2, which spread very quickly around the world, has created a threat to public health.

Qeadan et al. [7] pointed out that during the SARS epidemic, the percentage of complications and deaths among pregnant women was significantly higher than usual.

Schwartz and Dhaliwal said that virus transmission from the mother to the foetus led to miscarriage or development of foetal malformations or foetal death [8]. Taking into account the possibility of occurrence of vertical transmission of infection from the mother to the foetus or newborn, who has an immature immune system, pregnant women and newborns should be regarded as a potential risk group [9].

The aim of our publication was to analyse the available literature in terms of the impact of COVID-19 on the course of pregnancy and the health of the newborn.

## 2. Main Text

### 2.1. Materials and Methods

The articles from prestigious scientific journals covering the period from 2020 to February 2022 and relevant review papers and original research articles from PubMed were analysed.

In order to analyse the available research literature, the Web of Science, Scopus, and PubMed databases were used, in which the search for publications was conducted using keywords (“pregnancy,” “coronavirus,” “SARS-CoV-2,” and “newborn”). Analysis of clinical trials and randomized controlled trials was additionally performed using PRISMA (Preferred Reporting Items for Systemic Reviews and Meta-Analysis) guidelines (flowchart), according to inclusion and exclusion criteria, full-text English, and relation to infection of COVID-19 and pregnancy. Meta-analyses and systematic reviews (2022–2023) on symptoms, neonatal course, and risk of COVID-19 infection have been summarized (Table 1). Summary of meta-analyses and systematic reviews (2022–2023) on the effect and adverse reaction of the COVID-19 vaccination is presented in Table 2.

### 2.2. Selection Criteria

**Figure 1:**
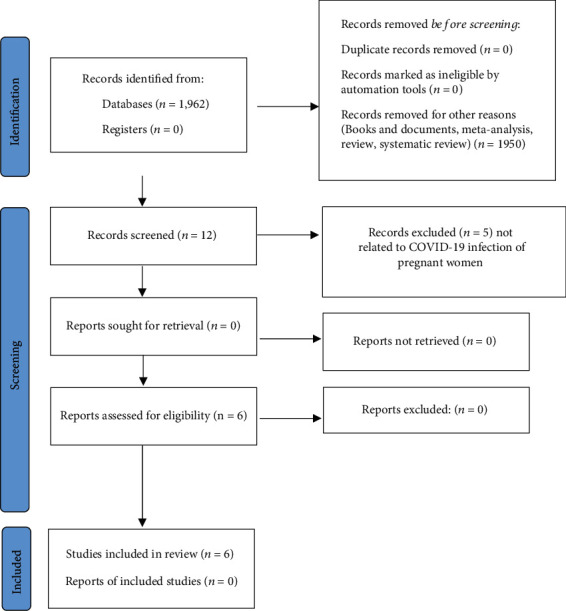
Flowchart presenting the study selection according to PRISMA guidelines.

Figure 1 is a flowchart presenting the selection of randomized controlled trials and clinical trials according PRISMA guidelines.

## 3. Results and Discussion

In Table 3 below, we present the summary of the randomized controlled trials and clinical trials in accordance with PRISMA.

In 2020, the College of American Pathologists studied 38 pregnant women and their neonates. The women analysed had SARS-CoV-2 infection during pregnancy. The clinical data and results of laboratory tests and virological tests were taken into account. The studies revealed that no transmission of the virus from mother to child had occurred in any of the women who had suffered the infection. None of the examined swabs taken from the newborns and none of the placentae examined in some cases suggested any intrauterine or transplacental transmission of the SARS-CoV-2 from the women to their foetuses [46]. The clinical trial (2021) analysed the placentas of 31 COVID-19-positive mothers via reverse transcriptase PCR, immunohistochemistry, and in situ hybridization. Only one case of placental infection was detected, which was associated with intrauterine demise of the foetus [43].

The study by Khoury et al. [46] conducted in five New York hospitals in 241 female patients revealed that almost 30% of COVID-19 cases had a severe or critical course. Most pregnancies in those patients were terminated via caesarean sections. The result of the study correlated with overweight, determining the severity of the infection course. Women with a BMI value of 30 or more had a more severe course of the infection.

Jamieson and Rasmussen presented their data, suggesting that pregnant women with COVID-19 three times more frequently required hospitalisation in an intensive therapy unit, compared with nonpregnant females. Pregnant women with COVID-19 almost three times more frequently required mechanical ventilation, and their mortality rate was 1.7 times higher [47]. Obese women, with a BMI of 30 or more, had a more severe course of COVID-19, and their infection intensified. Ferrugini et al. [41] in clinical trials suggest that it is important to test SARS-CoV-2 infections during pregnancy to prevent complications. A total of 265 pregnant women were included in the study. Patients exposed or infected with SARS-CoV-2 had a higher incidence of preterm delivery, caesarean section, and need for resuscitation in the delivery room. The results showed a high rate of positive tests among newborns (37.5%).

Carrasco et al. conducted a study in Spain in 105 pregnant women with COVID-19 and found that the rate of prematurity reached over 20%, and almost 17% of the babies required hospitalisation in an intensive therapy unit [48].

In the period between 1^st^ March and 14^th^ April 2020, Knight et al. [49] conducted a study in 194 centers located in the United Kingdom, including an analysis of 427 females admitted to hospitals with SARS-CoV-2 infection. In the study, the UK Obstetric Surveillance System (UKOSS) was used. The authors observed that 233 (56%) pregnant women requiring hospitalisation were black or belonged to another ethnic minority. Again, attention was paid to the fact that 281 women, i.e., 62% of the study population, were overweight. Therefore, overweight correlated with COVID-19. Over 40% of cases were women aged 35 years and older, and 145 (34%) had comorbidities. In most women (196, i.e., 73%), the pregnancies could have been terminated with labour at term. Unfortunately, five women, i.e., 1%, died during COVID-19, and 41, i.e. 10%, required respiratory support. Carrasco et al. also reported that 5% of 256 neonates proved positive in the test for SARS-CoV-2 within 12 hours after birth [48].

Wang et al. [50] demonstrated in their study that SARS and MERS contributed to miscarriages, intrauterine deaths, and even foetal growth restriction and a high mortality rate. The authors confirmed that pneumonia caused by COVID-19 in pregnant patients had a similar course to that occurring in nonpregnant females. It was also found that pregnant women were not at a higher risk of acquiring COVID-19 and of its more severe course. Furthermore, Wang et al. [50] have reported that currently no evidence is available that the virus can be transmitted to the foetus during pregnancy or labour. The authors also observed that infants and babies only experienced mild forms of COVID-19. At the same time, they recommended further studies on the effect of COVID-19 on pregnancy course and foetal development [50].

The analysis of the data and reports concerning the role of placenta, vertical transmission in SARS-CoV-2 infection, and results of prenatal exposure to the virus were described in the paper by Prochaska et al. [51]. According to the opinion presented in the publication and observations by other authors [52], the effect of SARS-CoV-2 on the developing foetus remains not fully elucidated. The presented case reports, analysed by Prochaska et al. [51], suggest that the transmission from mother to foetus is rare. Some evidence is, however, available that suggests that a SARS-CoV-2 infection of the placenta and foetus is possible. Infected placentae show inflammatory, thrombotic, and vascular changes. The authors stressed that the inflammatory character of SARS-CoV-2 infection during pregnancy can cause obstetric and neonatal complications. The exposure to intrauterine inflammation and placental lesions can also potentially lead to long-lasting multisystem defects in the neonates, the mothers of whom had COVID-19 [52].

On the other hand, the study by Schwartz [52] demonstrated no fatal cases of COVID-19 in pregnant women. In the study, no virus transmission from infected mothers to foetuses was found. Importantly, similar to the case of pregnancies with SARS and MERS infections, no confirmed cases were reported of intrauterine SARS-CoV-2 transmission from mothers with COVID-19 to their foetuses. All studied samples from the neonates, in some cases including the placentae, proved SARS-CoV-2-negative on RT-PCR. At this moment of the COVID-19 global pandemic, there is no evidence that SARS-CoV-2 intrauterine or transplacental transmission from infected pregnant women to their foetuses is possible. The authors recommend further studies in this respect [46, 52].

The pathogenetic process, aspects of pathology, and clinical data concerning COVID-19 with particular consideration of pregnancy and its course and, moreover, histological analysis of the placentae of pregnant women infected with SARS-CoV-2 compared with those infected with SARS-CoV and MERS-CoV were presented by Wenling et al. [3]. Based on a literature review, the authors have confirmed that the mentioned viruses cause a cytokine storm in the body, an extensive immune response, and cause changes in peripheral lymphocytes and immune system cells, leading to pregnancy complications resulting from that infection. The expression of ACE2 receptors in vascular endothelium may correlate with histological changes in the placentae of pregnant women infected by SARS-CoV-2. No unequivocal evidence is available as yet to support foetal infection through intrauterine vertical transmission of SARS, MERS, and SARS-CoV-2 viruses, but many reports confirm and point out the risk of death of mothers due to COVID-19. Therefore, pregnant women and newborns require special care in respect of COVID-19 prophylaxis, diagnosis, and treatment [3].

Caparros-Gonzalez [53] in his review paper concludes that pregnant women show no serious signs after getting COVID-19, and according to the observations of Wenling et al. [3], pregnant women with pneumonia in the course of COVID-19 show clinical characteristics similar to those of nonpregnant women. Caparros-Gonzales [53] demonstrated however, that neonates were at a higher risk of complications, and the course of COVID-19 was more severe, posing a threat to the neonate. In the analysed review material, the case was described of a premature infant, in the mother of whom a COVID-19-induced pneumonia was diagnosed, and that infection led to death of the baby. It was not confirmed, however, that a vertical transmission occurred from the mother to the baby. In his literature review, Caparros-Gonzalez [53] concluded that COVID-19 seemed to be milder for pregnant women than for their newborns.

Transmission of SARS-CoV-2 from the mother to foetus has not been confirmed in the study by Naidu et al. in 2022. According to their observations, the absence of transmission from the mother to foetus may by associated with the presence of lactoferrin in the placenta, amniotic fluid, and milky discharge. The authors stress the key role of lactoferrin in ensuring immunity. The cytokine storm caused by COVID-19 in pregnant women can produce significant damages, such as foetal inflammatory conditions, and, if not controlled, may later result in disorders from the autism spectrum and abnormalities of brain development in newborns. Taking into account this significant threat to a child's growth and development, the prevention of COVID-19 during pregnancy should be given a high priority [54].

Kyle et al. [55] observed that neonates born to mothers infected by SARS-CoV-2 rarely acquired the disease or presented unfavourable clinical results. At the same time, the authors have stressed that the COVID-19 pandemic is still spreading worldwide and that the SARS-CoV-2 virus will stay with the human population; therefore, it is important to identify the populations at risk and to establish an adequate strategy of clinical care in order to protect the human population and, in particular, the newborns. Burwick et al. [40], in clinical trials they conducted, describe the use of eculizumab to treat severe COVID-19 in a small series of pregnant and postpartum adults. There were no serious adverse maternal or neonatal events attributed to eculizumab at 3 months. In order to protect the human population, and in particular newborns, Friedman et al. [56] conducted their research. Data provide reassurances that the maternal use of hydroxychloroquine is associated with a low incidence of infant QTc prolongation. However, if included in clinical COVID-19 studies, early postnatal ECGs should be considered.

The observations by Kyle et al. [55] are in concordance with those reported by Salem et al. [9], who said that infections of pregnant women diagnosed with COVID-19 were usually asymptomatic or had mild or moderate symptoms. They concluded that pneumonia is one of the most frequent conditions in pregnant women with COVID-19. It cannot be unequivocally said, however, that SARS-CoV-2 infection increases the risk of maternal, foetal, and neonatal complications [55]. The COVID-19 pandemic represents a collective trauma that may have enduring stress effects during sensitive periods, such as pregnancy. Prenatal stress may result in epigenetic signatures of stress-related genes (e.g., the serotonin transporter gene, SLC6A4) that may in turn influence infants' behavioral development [42]. Based on inhibition of viral replication and limited reports on clinical efficacy, hydroxychloroquine (HCQ) is being considered as prophylaxis and treatment of COVID-19 [56]. These data provide reassurances that the maternal use of HCQ is associated with a low incidence of infant QTc prolongation. However, if included in clinical COVID-19 studies, early postnatal ECGs should be considered.

On the other hand, Papapanou et al. [57] observed an increased incidence of caesarean sections and premature births in women with COVID-19.

Oncel et al. [45], in clinical trials they conducted, stated if COVID-19 in pregnant women has important impacts on perinatal and neonatal outcomes. Maternal mortality, higher rates of preterm birth and caesarean section, suspected risk of vertical transmission, and low rate of breastfeeding show that family support should be a part of the care in the NICU. In the case of symptomatic women (COVID-19) with confirmed infection, a high percentage was reported of admissions of mothers and neonates to ITUs. Papapanou et al. [57], similar to Salem et al. [9], suggest that a possibility of vertical transmission from the mother to baby cannot be ruled out. The authors of the publications recommend further studies in pregnant women during the COVID-19 pandemic [9, 57]. In the study by Ayed et al. [58], two cases have been presented of neonates born spontaneously through natural passages after premature rupture of the membranes, to mothers with positive results of RT-PCR tests. The babies were born in the 31^st^ and 39^th^ weeks of pregnancy, and during the birth of the first of them, a fever occurred in the mother, and then, the COVID-19 infection was confirmed [58].

The literature review shows that a vertical transmission from the mother to baby is possible. The third trimester seems to be the period of key importance and the highest sensitivity in the course of the infection. The authors recommend further studies to activate surveillance programs at the end of the second trimester. Both Salem et al. [9] and other authors suggest that during this pandemic, a monitoring of pregnant women before and after labour and of their infants is necessary [3, 51, 55].

Elsaddig and Khalil [59] have found, however, that pregnant women with COVID-19 in the third trimester are more likely than their nonpregnant counterparts to require intensive care, though this may reflect a lower threshold for intervention in pregnant women rather than more serious disease [59]. Outcomes of neonates born to COVID-19-positive women are generally very good, though iatrogenic preterm births are more common. Elsaddig and Khalil suggest a need of further monitoring of the results of pregnant women and those directly after labour, during the pandemic [59].

In the study by Ko et al. [60], the data from 703 hospitals were considered. Out of 489,471 obstetric hospitalisations, in 6550 cases (1.3%), COVID-19 was diagnosed. Moreover, in the women with COVID-19, the following were observed: respiratory failure, deaths, sepsis, and shock. Women diagnosed with COVID-19 were more frequently admitted to intensive care units. Furthermore, in such pregnant women, acute renal failure, thrombosis in the placental chorion, thromboembolism, and premature births were observed. In view of that, the authors recommended implementation of prophylactic strategies in order to reduce the risk of severe acute respiratory failure after COVID-19 [60–62].

Mirbeyk et al. [63] in their literature review considered 37 papers describing 364 pregnant women with COVID-19 and 302 newborns and found that a definite majority of the pregnant women were in the third trimester of pregnancy, and only 45 pregnant patients had COVID-19 in the first or second trimester of pregnancy (12.4%). In most mothers, mild-to-moderate symptoms of COVID-19 occurred. Out of 364 pregnant women, 25 were asymptomatic on admission. The most frequent signs included fever (62.4%) and cough (45.3%). Two mothers with COVID-19 died. Some pregnant patients (12.1%) had negative results of tests for SARS-CoV-2, but they presented COVID-19-related clinical signs and abnormalities on computed tomography (CT) examinations. Severe pneumonia developed in 22 (6.0%) pregnant patients. The two cases of death of the mothers were due to severe pneumonia and multiple organ dysfunction. The study involved the total number of 302 neonates born to mothers with COVID-19. It was found that 65 out of 302 newborns were premature babies, what accounted for 23.6%. Death was reported of one baby, born to a mother with COVID-19 (a stillbirth). Out of the babies born to mothers with COVID-19, five neonates were in critical condition and two neonates later died. Mirbeyk et al. [63] concluded that their systematic review of published studies confirmed that the course of COVID-19 in pregnant women was similar to that in other populations. No sufficient data are available, however, to say that COVID-19 poses a risk for the developing pregnancy [63].

In order to prevent COVID-19 complications, Wainstock et al. [64] conducted studies in pregnant women, confirming the effectiveness of vaccinations. They studied the association between prenatal Pfizer-BioNTech COVID-19 vaccination and pregnancy. It was found that prenatal maternal COVID-19 vaccine had no adverse effects on pregnancy course and outcomes. Clinical trial findings of Cosma et al. [44] may help to guide the global COVID-19 vaccination program in pregnancy. In sequential samples collected up to at least 6 months after SARS-CoV-2 infection in pregnant patients, we detected a typical antibody response after an acute viral infection. In those who developed a neutralizing antibody response, the titers were maintained for the entire length of pregnancy and transmitted to the newborn [44].

Pregnant women are one of the vulnerable groups that require special care during the COVID-19 epidemic (20). They are, therefore, more susceptible to infectious diseases such as SARS-COV-2 (5).

The published observations were analysed by comparing the results of mothers and pregnancies, perinatal results, and results of newborns and infants from the time of the pandemic, comparing them to the period before the pandemic period [14, 28].

The infection, symptoms, infection severity, the need for intensive therapy, and mechanical ventilation were analysed, as well as childbirth and puerperium of women with COVID-19 infection [14, 18].

The number of premature births (PTB), birth weight, and maternal/foetal mortality was determined [10, 11, 13, 16, 20, 28].

Additionally, the aim of the study was to determine SARS-CoV-2 infection in children born to SARS-CoV-2-infected mothers, time of transmission from the mother to child, and perinatal results [47, 65].

Despite the increasing number of studies on the risks of COVID-19, the assessment of the impact of the pandemic on childbirth and preterm birth remains contradictory. Perinatal outcomes and reports are also conflicting [19]. Current data suggest little or no potential for vertical transmission of COVID-19 from pregnant women to the foetus during pregnancy or delivery. Controversies are evident in the quality and design of published research.

The COVID-19 pandemic has had a significant impact on healthcare systems and on women's health as well as their mental state. The results from all observations and published works prompted us to collect and systematize the data (Tables 1 and 2) from publications created until 03.2023.

This review additionally discusses the results and conclusions important in terms of prevention and, thus, vaccination against COVID-19 infection. The prevention of COVID-19, as we have shown above, is crucial to ensure the health of the mothers and newborns. Knowledge, awareness, and, thus, the attitude and practice of pregnant women, staff, and all society towards COVID-19 infection are fundamental.

In Table 2 below, we present the collected data related to not only effectiveness but also the potential risk of vaccination or side effects.

## 4. Conclusions

Pregnant women, foetuses, and neonates are possibly the population at high risk during the present coronavirus disease 2019 (COVID-19) pandemic caused by the SARS-CoV-2 virus. The number of publications on the consequences of COVID-19 during pregnancy is slowly growing, and for the sake of the mothers and neonates, an analysis of that issue is, by all means, justified. A SARS-CoV-2 infection can disturb female reproductive system functions, leading to menstrual cycle disorders, complications of pregnancy development, or miscarriages [66, 67]. Most literature reports suggest that the course of the infection in pregnant women is similar to that in the general population [68]. However, some women require a respiratory support, and they are also at a risk of death [69]. The reports are alarming that a significant percentage of pregnancies is terminated prematurely by caesarean section. Such delivery by caesarean section leads to a need of care of the neonate, who is at a higher risk of complications. Newborns have a poorer intestinal flora, which, as it is known, is the foundation of the normal immunity of the newborn's organism. Although SARS-CoV-2 infection was found in few neonates, a transmission of the virus from the mother to foetus cannot be ruled out [70, 71]. The long-term developmental consequences for babies of mothers who proved virus-positive during pregnancy are only the subject of research. Further studies and follow-up will make an assessment of the long-term consequences possible.

## Figures and Tables

**Table 1:** Summary of the meta-analyses and systematic reviews (2022–2023) concerning the symptoms, neonatal outcome, and risk of COVID-19 infection.

Year of study/the country of study	Authors	Number of studies/pregnant women and children	Symptoms of women	Neonatal outcomes of children	Main conclusion
2022/Brazil	de Medeiros et al. [10]	70 studies 10,047 pregnant women with COVID-19 (71.6%), third trimester maternal mortalities (2%), and abortions (5%).	The most common symptoms: fever, cough, chest pain, dyspnea, and fatigue Delivery: preterm (24%) and caesarean (42%).	The neonatal outcomes: foetal distress (11%), birth weight (15%), Apgar < 7 (19%), the neonatal intensive care unit (28%), and foetal mortality (2%).	No evidence of severe acute respiratory syndrome coronavirus-2 in the placenta, breast milk, umbilical cord, and amniotic fluid of pregnant patients.

2022/USA	Smith et al. [11]	21 studies, 33 countries and territories, and 21,977 cases of SARS-CoV-2 infection in pregnancy or postpartum.	Increased risk for COVID-19 severity maternal morbidities, adverse pregnancy outcomes, less common risk factors (HIV infection, prepregnancy underweight, and anemia) HIV 1.74 times intensive care unit Underweight before pregnancy at higher risk of ICU (RR, 5.53) Ventilation (RR, 9.36) Pregnancy-related death (RR, 14.10) Prepregnancy obesity Severe COVID-19 outcomes: intensive care (RR, 1.81), ventilation (RR, 2.05), and critical care (RR, 1.89), pneumonia (RR, 1.66) Anemic pregnant women with COVID-19: risk of intensive care unit (RR, 1.63) and death (relative risk, 2.36).	COVID-19-increased risk of foetal death, low birthweight for women with comorbidities (preexisting diabetes mellitus, hypertension, and cardiovascular disease).	Pregnant women considered as a high-risk population Special priority for prevention and treatment for pregnant women with additional risk factors.

2022/Iran	Pashaei et al. [12]	44 articles 2375 women with COVID-19 (second, third trimester of pregnancy), 1725 deliveries, 2716 newborns (foetal).	The most common symptoms: mild or moderate pneumonia, no comorbidity (73%), fever (19%), cough (17%), pulmonary changes (7.5%), increased CRP (8%), lymphocytopenia (9.4%) Delivery method: C-section delivery (913, 53%), normal vaginal delivery (812, 47%).	13 died (5 with the mother) 118 SARS-CoV-2/1965 tested Vertical transmission 7/145 tested.	13 died (5 with the mother) 118 SARS-CoV-2/1965 tested Vertical transmission 7/145 tested.

2022/Canada	Yang et al. [13]	52 studies 2,372,521 pregnancies (the pandemic period) 28,518,300 pregnancies (prepandemic period).	Significant reduction (only uaOR 0.95) of PTB (43 studies) For single centers/health areas (29 studies) Not in regional/national studies (14 studies, uaOR 0.99) Reduction in spontaneous PTB (9 studies) No difference of stillbirth (pandemic/prepandemic periods (32 studies, OR 1.18) Increased maternal mortality (5 studies, uaOR 1.15) Significant publication bias for the outcome of PTB.	An increase (mean) pandemic birthweight (9 studies, mean difference 21 g).	No statistically significant difference in stillbirths between pandemic and prepandemic periods The COVID-19 pandemic may be associated with a reduction in PTB (referral bias cannot be excluded).

2022/Turkey	Karaçam et al. [14]	54 studies 517 pregnant with COVID-19 385 infants.	Delivery: preterm labour (18%), caesarean (77%), maternal mortalities (9).	Newborns: low birth weight (19%), foetal distress (14%), neonatal intensive care unit (24%), baby mortalities (8).	COVID-19 in pregnant women—negative maternal and infant outcomes, with mortalities.

2022/UK	Allotey et al. [15]	472 studies (206 cohort studies and 266 case series and case reports) 28,952 mothers 18,237 babies 40 studies (1.8%) COVID-19-positive babies/14,271 COVID-19-tested mothers.	Severe maternal infection (OR 2.4) Maternal death (14.1) Intensive care unit (3.5) Maternal postnatal infection (5.0) associated with SARS-CoV-2 positivity in babies Positivity rates different between regions: North America (0.1%), Latin America and the Caribbean (5.7%).	Early pregnancy losses (8) SARS-CoV-2-positive babies: Stillbirths (20/800) Neonatal deaths (23/800) Alive at the end of follow-up (749/800) 592/14,271 SARS-CoV-2 babies with positive mother-to-child transmission (14) In utero (7/448) In intrapartum (2/18) During the early postnatal period (5/70).	SARS-CoV-2 positivity rates low in babies born to mothers with SARS-CoV-2 infection confirmed vertical transmission of SARS-CoV-2 (likely rare) Severity of maternal COVID-19 appears to be associated with SARS-CoV-2 positivity.

2022/China	Yao et al. [16]	63 reports 3,220,370 pregnancies (COVID-19 pandemic) 6,122,615 pregnancies (prepandemic).	Significant decrease: Preterm birth: PTB < 37 weeks′ gestation (OR 0.96; 78.7%; 62 studies) PTB < 28 weeks′ gestation (OR 0.92; 26.4%; 25 studies) Borderline significant reduction in the odds of very PTB (<32 weeks' gestation; pooled OR [0.86, 1.01]; 33 studies) pre-/pandemic Significant publication bias for PTB.		COVID-19 pandemic associated with preterm birth Only a borderline significant reduction for very PTB during the pandemic compared with the prepandemic period Conflicting results, further research on whether the change is related to pandemic mitigation measures was warranted.

2022/China	Luo et al. [17]	102 systematic reviews 68 studies (66.7%) of high quality, 19 studies (18.6%) of medium quality, and 15 studies (14.7%) of low quality 74 (72.5%) general populations 15 (14.7%) neonates, children, and adolescents 13 (12.8%) pregnant women.	Identification of 74 COVID-19 symptoms: respiratory system (17), neurological system (21), gastrointestinal system (10), cutaneous symptoms (16), ocular symptoms (10) The most common symptoms (prevalence): fever (67 studies, 64.6%), cough (68 studies, 53.6%), muscle soreness (56 studies, 18.7%), fatigue (52 studies, 29.4%).		The prevalence for COVID-19 symptoms generally lower in neonates, children and adolescents, and pregnant women than in the general populations; at least 74 different clinical manifestations associated with COVID-19 Attention should also be paid to the rare symptoms to help in the early diagnosis of the disease.

2022/Iran	Simbar et al. [18]	141 studies 1,843,278 pregnant women 74 cohort and case-control studies.	Pregnancy outcomes: preterm delivery, maternal mortality, NICU admission, pregnancy loss.	Death of SARS-CoV-2-positive neonates for lower middle income is higher than that for high income.	Vertical transmission from mother to foetus which may result in immediate and long-term effects on the newborn is unclear.

2022/UK	Hawco et al. [19]	38 studies.	No overall effect of mitigation measures against COVID-19 on preterm birth < 37 weeks (OR 0.96) Reduction in preterm birth: <37 weeks (OR 0.89), <34 weeks (OR 0.56), for iatrogenic births in singleton pregnancies Significant reduction in preterm births: <34 weeks (OR 0.71), no effect on risk of stillbirth, birth weight NICU significantly reduced (OR 0.87).		The reduction in preterm births in regions with high mitigation measures against SARS-CoV-2 infection likely driven by a reduction in iatrogenic births.

2022/Poland	Pilarska et al. [20]		COVID-19 pregnant women: caesarean delivery (80%), preterm delivery (26%), premature rupture of the membranes (9%), stillbirth (2%), premature delivery (25%), SARS-CoV-2 mainly in syncytiotrophoblast cells at the maternal-foetal border of the placenta Histological examination of the placenta-dense macrophage infiltration Abnormal perfusion of foetal blood vessels or foetal vascular thrombosis The umbilical cord blood-elevated levels of SARS-CoV-2 IgG or IgM (9 newborns), evidence of vertical infection.	Neonatal complications: foetal distress (8%), respiratory distress syndrome (8%), pneumonia (8%), deaths (4 newborns).	The features of placental damage in women with COVID-19 clearly different from the control group Further research needed to better understand how SARS-CoV-2 infection affects the placenta.

2022/Canada	Jeganathan and Paul [21]	47 studies 11,88 SARS-CoV-2-positive pregnant women 985 neonates.	Vertical transmission (0.3%, 3): probable (0.5%, 5), possible (1.8%, 17), unlikely (80.3%, 724), not infected (17%, 153).		

2022/China	Meng et al. [22]		Intrauterine SARS-CoV-2 infection affects the auditory system of the newborn due to intrauterine hypoxia and vertical transmission.	Possibility of SARS-CoV-2 influence on hearing loss (HL) in newborns during the second and third trimesters of pregnancy.	All newborns whose mothers had COVID-19 during pregnancy should be evaluated for cochlear function, regardless of symptoms of COVID-19 of their mothers. In the COVID-19 era, newborns should be provided with audiological evaluation including teleaudiology.

2022/SAU	Aljohani et al. [23]	27 studies (22/China, 1/US, 1/Honduras, 1/Italy, 1/Iran, 1/Spain), 386 pregnant women with COVID-19, 334 newborns.	Already delivered (356/386), medical abortions (4), still pregnant (28), died before delivery from COVID-19 (2), caesarean sections (71%) Common symptoms: fever and cough, premature rupture of membranes, distress, and preterm birth pregnancy complications The common: lymphopenia, leukocytosis, elevated levels of CRP Abnormal CT viral lung changes (73.3%).	Infants tested positive for severe acute respiratory syndrome coronavirus 2 (SARS-CoV-2) infection (11) Common outcomes for newborns: low birth weight, short gestational age, most infants with lymphopenia and thrombocytopenia.	The evidence of adverse pregnancy and neonatal outcomes caused by COVID-19 The clinical features of pregnant women similar to those of generally infected patients No evidence of vertical transmission.

2022/India	Panda et al. [24]	14 studies of India 3551 neonates 3542 SARS-CoV-2-positive mothers.	The primary outcomes: delivery, perinatal asphyxia, preterm birth, breastfeeding The pooled rates of premature birth (18.89%), caesarean delivery (55.89%), breastfeeding (67.79%), neonatal mortality, 12.64/1000 live births.	The primary outcomes: breastfeeding, neonatal mortality, SARS-CoV-2 infectivity among neonates of SARS-CoV-2 mothers SARS-CoV-2 positivity rate (5.28%) Symptomatic (11.76%) SARS-CoV-2-positive neonates who died (5/281; 1.7%) Indian neonatal of SARS-CoV-2-positive mothers (~5%) COVID-19 infected, majority—good clinical outcomes Lower mortality among neonates born to SARS-CoV-2-positive mothers compared to Indian baseline	

2023/USA	Smith et al. [25]	12 studies 12 countries 13,136 pregnant women.	Significantly increased risk: maternal mortality (10 studies; 1490; RR 7.68), admission to intensive care unit (8 studies; 6660; RR 3.81), mechanical ventilation (7 studies; 4887; RR 15.23), receiving any critical care (7 studies; 4735; RR 5.48), diagnosed with pneumonia (6 studies; 4573; RR 23.46), thromboembolic disease (8 studies; 5146; RR 5.50), preterm (7 studies; 6233; RR 1.71), moderately preterm (7 studies; 6071; RR 2.92).	Born with low birth weight (12 studies; 11 930; RR 1.19) Infection not linked to stillbirth Neonates born to women with SARS-CoV-2 Neonatal care unit after birth (7 studies; 7637; RR 1.86).	SARS-CoV-2 infection at any time during pregnancy connected with the risk of maternal death, severe maternal morbidities, and neonatal morbidity, but not stillbirth or intrauterine growth restriction.

2022/UK	Sheikh et al. [26]	311 studies 57 countries 2,003,724 pregnant women.	Lower-middle-income countries significantly higher rates: maternal mortality (0.68%; 3 studies, 31,136 women), intensive care admission (4.53%, 54 studies, 23,420 women), stillbirths (1.09%; 41 studies, 4724 women) than high-income countries COVID-19 complications disproportionately affected South Asia (highest maternal mortality 0.88%, 17 studies, 2023 women) and Latin America and the Caribbean (the highest stillbirth rates 1.97%; 10 studies, 1750 women).		The rates of SARS-CoV-2 infection in pregnant women significantly different between regions (highest rates are Latin America and the Caribbean (19%, 13 studies, 38,748 women) and lower-middle-income countries (13%, 25 studies, 100,080 women)) Health outcomes mirror the COVID-19 burden and global maternal and offspring inequalities.

2022/China	Deng et al. [27]	18 studies 133,058 SARS-CoV-2 infection during pregnancy 99,567 cases of SARS-CoV-2 wild type or prevariant infection 33,494 cases of SARS-CoV-2 variant infections.	Among pregnant women with SARS-CoV-2 infections, delta period: respiratory support (27.24%), severe or critical illness (24.96%), intensive care unit (ICU) admission (11.31%), maternal death (4.20%), preterm birth <37 (33.85%) Pre-delta period: respiratory support (10.74%), severe or critical illness (10.74%), intensive care unit (ICU) admission (4.17%), maternal death (0.63%), preterm birth < 37 (18.58%) Omicron period: respiratory support (2.63%), severe or critical illness (1.11%) ICU admission (1.83%) lower than in the pre-delta and delta periods.		Omicron infections associated with less severe maternal and neonatal adverse outcomes, though maternal ICU admission, the need for respiratory support, and preterm birth did also occur with omicron infections Omicron currently the predominant strain, highest rates of transmission, should not be ignored, adverse risks of maternal ICU admission, respiratory support, preterm births in pregnant patients with SARS-CoV-2 infections, health of mothers and infants should be protected

2022/Canada	Yang et al. [28]	45 studies 1,843,665 pregnancies/pandemic period 23,564,552 pregnancies/prepandemic period.	Significant reduction of PTB (35 studies, uaOR 0.95), (6 studies, aOR 0.95) The reduction in single centers/health areas (25 studies, uaOR 0.90) Not in regional/national studies (10 studies, uaOR 0.99) Reduction in spontaneous PTB (6 studies, uaOR 0.89) Induced PTB (5 studies, uaOR 0.89) No difference of stillbirth pandemic/prepandemic (24 studies, uaOR 1.11) (4 studies, aOR 1.06) Maternal mortality increased (4 studies, uaOR 1.15).	An increase in birthweight (mean) in the pandemic period) (6 studies, mean 17 g).	No statistically significant difference in stillbirth (pandemic vs. prepandemic) The COVID-19 pandemic potentially associated with a reduction in PTB (referral bias cannot be excluded).

2022/France	Aho Glele et al. [29]				Infection during pregnancy associated with preterm may be associated with preeclampsia; more data is needed for stillbirth.

**Table 2:** Summary of the meta-analyses and systematic reviews (2022–2023) concerning the effects and adverse effects of COVID-19 vaccination.

Year of study/the country of study	Authors	Number of studies/pregnant women/children	Publication aim	Results	Adverse effects
2022/UK	Prasad et al. [30]	23 studies 117,552 COVID-19-vaccinated pregnant women.	Assessment of the safety and effectiveness of COVID-19 vaccines during pregnancy.	(1) Effectiveness (89.5%) of mRNA vaccination (2) Reduction in stillbirth (the risk significantly lower by 15% mRNA vaccination in pregnancy appears to be safe).	No evidence of a higher risk of adverse outcomes (miscarriage, earlier gestation at birth, placental abruption, pulmonary embolism, postpartum hemorrhage, maternal death, intensive care unit admission, lower birthweight).

2022/USA	Rawal et al. [31]	32 studies Pfizer, Moderna (24) Janssen (6).	Assessment of safety, immunogenicity, effectiveness, acceptance of COVID-19 vaccination among pregnant people in the United States.	11 examined COVID-19 vaccine safety, 10 investigated immunogenicity and effectiveness, 11 assessed vaccine acceptance among pregnant people COVID-19 vaccination, pregnant women with a robust immune response, and vaccinations conferred protective immunity to newborns through breast milk and placental transfer.	Injection-site pain and fatigue are the most common adverse events One case study showed immune thrombocytopenia COVID-19 vaccination did not increase the risk of adverse pregnancy or neonatal outcomes.

2022/Spain	Novillo and Martínez-Varea [32]	33 studies	Studying the role of/provide an update regarding COVID-19 vaccines during pregnancy, breastfeeding.	Reduction of the risk of severe COVID-19 in pregnant women after COVID-19 vaccination Induction artificial active immunogenicity in the mother and natural passive immunogenicity in the child Breastmilk straddles both immediate antibody-mediated and long-lived cellular-mediated immune protection Neonatal benefits Vaccination associated with a larger and more stable immunoglobulin G response COVID-19 infection associated with a rapid and long-lasting immunoglobulin A response Strong recommendation of COVID-19 vaccines for pregnant and breastfeeding populations to protect mothers and newborns.	Main adverse effect (pain at the injection site, as in the general population) Adverse effects more frequent after the second dose, slightly more frequent after the Moderna vaccine.

2022/Italy	Umberto De Rose et al. [33]	45 studies 74,908 pregnant vaccinated women/5098 lactating women.	Assessment of the current knowledge about maternal and neonatal outcomes following COVID-19 vaccination during pregnancy and breastfeeding Estimation of how many pregnant and lactating women were vaccinated and with maternal and neonatal outcomes.	Recommendation for vaccination against the SARS-CoV-2 virus for pregnant women Infants received specific SARS-CoV-2 antibodies after maternal vaccination Still limited evidence that fever during the first months of gestation increases the possibility of congenital anomalies, should be carefully counseled The same considerations apply to breastfeeding women, also considering the immune responses that mRNA vaccines can generate in their human milk.	No major side effects especially during the second and third trimesters of pregnancy and during breastfeeding.

2022/Norway	Magnus et al. [34]	157,521 singleton births (103,409 Sweden 54,112 Norway), 157,521 singleton pregnancies.	Estimation of the risk of adverse pregnancy outcomes after vaccination against SARS-CoV-2 during pregnancy identification of exposure and background characteristics mRNA vaccines: BNT162b2 (Pfizer-BioNTech), mRNA-1273 (Moderna), one vaccine-AZD1222 (AstraZeneca).	The risk of preterm birth and stillbirth was evaluated The risk of being small for gestational age, low Apgar score, and neonatal care admission was evaluated using logistic regression The mean maternal age at the time of delivery was 31 years, and 28,506 (18%) were vaccinated (12.9% BNT162b2, 4.8% mRNA-1273, and 0.3% AZD1222) while pregnant First (0.7%), second (8.3%), and third trimesters (9.1%) of individuals delivering Vaccination not significantly associated with increased risk of preterm birth (6.2 vs. 4.9 per 10,000 pregnancy days; aHR, 0.98) Stillbirth (2.1 vs. 2.4 per 100,000 pregnancy days; aHR, 0.86) Small for gestational age (7.8% vs. 8.5%; difference, -0.6%; aOR, 0.97), low Apgar score (1.5% vs. 1.6%; difference, -0.05%; aOR, 0.97; 95% CI, 0.87 to 1.08) Neonatal care admission (8.5% vs. 8.5%; difference, 0.003%; aOR, 0.97) In Sweden/Norway, vaccination during pregnancy, compared with no SARS-CoV-2 vaccination during pregnancy, was not significantly associated with an increased risk of adverse pregnancy outcomes The majority of the mRNA vaccines during the second and third trimesters of pregnancy.	

2022/India	Krishna et al. [35]		Evaluation of the safety and probable outcomes of COVID-19 vaccination in pregnant women.	COVID-19 vaccination in pregnant women not associated with significant health risks, increased adverse effects, or complications to the mother, developing foetus, or newborn compared to nonvaccinated pregnant women Vaccinated pregnant women showed a robust immune response against COVID-19 infection.	

2023/Iran	Shafiee et al. [36]	11 studies 756,098 pregnant women	Evaluation of the outcomes of women who received the COVID-19 vaccine during pregnancy.	SARS-CoV-2 exposure during pregnancy related to adverse effects for both the mother and the infant SARS-CoV-2 vaccination lowered the risk of symptomatic disease The rate of neonates with 5 min Apgar score ≤ 7 Pregnant mothers with preterm birth significantly lower among vaccinated groups No evident differences observed among vaccinated pregnant mothers.	COVID-19 vaccination not associated with adverse pregnancy and neonatal outcomes.

2022/Egypt	Hagrass et al. [37]	13 studies 56,428 patients	Assessment of the maternal and neonatal safety of the COVID-19 vaccine during pregnancy.	No statistically significant difference of SAR-CoV-2 vaccination on the risk of outcomes: miscarriage, length of maternal hospitalisation, puerperal fever postpartum hemorrhage, instrumental or vacuum-assisted delivery incidence of Apgar score ≤ 7 at 5 min, birth weight.	

2023/China	Ding et al. [38]	42 studies of COVID-19 vaccination during pregnancy 96,384 (73.9%) BNT162b2, 30,889 (23.7%) mRNA-1273, 3172 (2.4%) other types 23,721 (18.3%) first trimester, 52,778 (40.5%) second trimester, 53,886 (41.2%) third trimester.	Evaluation of the safety of COVID-19 vaccination during evaluation of the association of COVID-19 vaccination during pregnancy with adverse maternal and neonatal outcomes.	Reduced risks of stillbirth or neonatal death COVID-19 vaccination during pregnancy not associated with congenital anomalies, preterm birth, NICU admission, or hospitalisation with an Apgar score at 5 min < low birth weight miscarriage, caesarean delivery, or postpartum hemorrhage.	COVID-19 vaccination during pregnancy not associated with any of the adverse neonatal or maternal outcomes.

2023/Greece	Kontovazainitis et al. [39]	2947 studies 7 cohort studies 67,274 pregnant women.	Assessment of COVID-19 vaccination's efficacy and safety during pregnancy; comparison of the incidence of major maternal and neonatal outcomes between SARS-CoV-2-vaccinated and SARS-CoV-2-unvaccinated pregnant women.	Reduction by 43% of the rate of SARS-CoV-2 infections among vaccinated pregnant women SARS-CoV-2 vaccination in pregnant women effective and safe.	SARS-CoV-2 vaccines not associated with major maternal and neonatal adverse events.

**Table 3:** Summary of the randomized controlled trials and clinical trials in accordance with PRISMA.

Year of study/the country of study	Authors	Number of participants	Publication aim	Main publication findings
2022/USA	Burwick et al. [40]	Eight participants; six during pregnancy, two in the postpartum period.	Evaluate the use of eculizumab for treatment of severe COVID-19 in pregnant and postpartum adults.	The median number of doses of eculizumab: 2 (range 1–3); the median time to hospital discharge: 5.5 days (range 3–12). All participants were alive and free of mechanical ventilation at day 29. No serious adverse maternal or neonatal events attributed to eculizumab at 3 months.

2022/Brazil	Ferrugini et al. [41]	A total of 265 pregnant women included in the study: 38 (14.4%) PCR-positive cases during pregnancy, 12 (31.6%) on admission screening, 71 (27.2%) patients IgM- and/or IgG-positive, 86 (32.4%), and at least one positive test during pregnancy.	Analysis of clinical and obstetric outcomes of pregnant women assisted in a high-risk maternity hospital in Brazil in 2020.	The most frequently reported symptoms: runny nose, cough, loss of smell and taste, headache, and fever. Rate of asymptomatic infections: 35%; rate of severe or critical infections: 4.6%. Patients exposed or infected with SARS-CoV-2 had a higher incidence of preterm delivery, caesarean section, need for resuscitation in the delivery room, Apgar score < 7 at 5 min, admission to the neonatal intensive care unit, and jaundice. Newborns with at least one positive test had significantly greater need for phototherapy after delivery (*p* = 0.05).

2021/Italy	Provenzi et al. [42]	Data from 108 mother-infant dyads.	Assessment of the behavioral and epigenetic vestiges of COVID-19-related prenatal stress exposure in mothers and infants. COVID-19-related prenatal stress at birth. Analysis of SLC6A4 methylation in thirteen CpG sites in mothers and infants' buccal cells. Analysis of infants' temperament at 3 months of age.	Greater COVID-19-related prenatal stress significantly associated with higher infants' SLC6A4 methylation in seven CpG sites. SLC6A4 methylation at these sites predicted infants' temperament at 3 months.

2021/Belgium	Colson et al. [43]	The placentas of 31 women infected with COVID-19 in 2019.	Analysis of the placentas of coronavirus disease-positive mothers via reverse transcriptase PCR, immunohistochemistry, and in situ hybridization.	Only one case of placental infection detected, which was associated with intrauterine demise of the foetus.

2021/Italy	Cosma et al. [44]	17 of 164 pregnant women positive for COVID-19.	Study of the SARS-CoV-2 antibody profile in pregnancy, from infection in the first trimester of pregnancy to delivery.	The presence of the same antibodies in arterial cord blood of all the newborns of women who developed IgG antibodies. Knowledge on the longevity and type of SARS-CoV-2 antibody response.

2020/Turkey	Oncel et al. [45]	125 pregnant women with COVID-19-positive RT-PCR tests and their newborns.	The epidemiological and clinical characteristics of newborns of COVID-19-infected women.	Important impacts of COVID-19 in pregnant women, on perinatal and neonatal outcomes. Eight of 125 mothers (6.4%) admitted to an intensive care unit for mechanical ventilation, higher maternal mortality (4.8%), caesarean section (71.2%), prematurity (26.4%), and low-birthweight infant (12.8%). 86.4% of the newborns followed in isolation rooms in the NICU. 3.3% newborns with a positive RT-PCR test result (one neonate positive on the second day, two on the fifth day). Suspected risk of vertical transmission (deep tracheal aspiration during the intubation, and the possible role of maternal disease severity on the outcomes).

## Abbreviations

ITU: Intensive therapy unit.

## References

[1] Gregory K. D., Davidson E. (1999). Prenatal care: who needs it and why?. *Clinical Obstetrics and Gynecology*.

[2] Wade G. H., Herrman J., McBeth-Snyder L. (2012). A preconception care program for women in a college setting. *The American Journal of Maternal Child Nursing*.

[3] Wenling Y., Junchao Q., Xia Z., Ouyang S. (2020). Pregnancy and COVID-19: management and challenges. *Revista do Instituto de Medicina Tropical de São Paulo*.

[4] Dashraath P., Wong J. L., Lim M. X. (2020). Coronavirus disease 2019 (COVID-19) pandemic and pregnancy. *American Journal of Obstetrics and Gynecology*.

[5] Baczyński B. (2013). *Preparation of pregnancy*.

[6] Holing E. V. (2000). Preconception care of women with diabetes: the unrevealed obstacles. *The Journal of Maternal-Fetal Medicine*.

[7] Qeadan F., Mensah N. A., Tingey B., Stanford J. B. (2021). The risk of clinical complications and death among pregnant women with COVID-19 in the Cerner COVID-19 cohort: a retrospective analysis. *BMC Pregnancy and Childbirth*.

[8] Schwartz D. A., Dhaliwal A., Rezaei N. (2021). Coronavirus Diseases In Pregnant Women, The Placenta, Fetus, and Neonate. *Coronavirus Disease - COVID-19. Advances in Experimental Medicine and Biology, vol 1318*.

[9] Salem D., Katranj F., Bakdash T. (2021). COVID-19 infection in pregnant women: review of maternal and fetal outcomes. *International Journal of Gynaecology and Obstetrics*.

[10] de Medeiros K. S., Sarmento A. C., Costa A. P. (2022). Consequences and implications of the coronavirus disease (COVID-19) on pregnancy and newborns: a comprehensive systematic review and meta-analysis. *International Journal of Gynaecology and Obstetrics*.

[11] Smith E. R., Oakley E., Grandner G. W. (2023). Clinical risk factors of adverse outcomes among women with COVID-19 in the pregnancy and postpartum period: a sequential, prospective meta-analysis. *American Journal of Obstetrics and Gynecology*.

[12] Pashaei Z., SeyedAlinaghi S., Qaderi K. (2022). Prenatal and neonatal complications of COVID-19: a systematic review. *Health Science Reports*.

[13] Yang J., D’Souza R., Kharrat A., Fell D. B., Snelgrove J. W., Shah P. S. (2022). COVID-19 pandemic and population-level pregnancy and neonatal outcomes in general population: a living systematic review and meta-analysis (update#2: November 20, 2021). *Acta Obstetricia et Gynecologica Scandinavica*.

[14] Karaçam Z., Kizilca-Çakaloz D., Güneş-Öztürk G., Çoban A. (2022). Maternal and perinatal outcomes of pregnancy associated with COVID-19: systematic review and meta-analysis. *European Journal of Midwifery*.

[15] Allotey J., Chatterjee S., Kew T. (2022). SARS-CoV-2 positivity in offspring and timing of mother-to-child transmission: living systematic review and meta-analysis. *BMJ*.

[16] Yao X. D., Zhu L. J., Yin J., Wen J. (2022). Impacts of COVID-19 pandemic on preterm birth: a systematic review and meta-analysis. *Public Health*.

[17] Luo X., Lv M., Zhang X. (2022). Clinical manifestations of COVID-19: an overview of 102 systematic reviews with evidence mapping. *Journal of Evidence-Based Medicine*.

[18] Simbar M., Nazarpour S., Sheidaei A. (2023). Evaluation of pregnancy outcomes in mothers with COVID-19 infection: a systematic review and meta-analysis. *Journal of Obstetrics and Gynaecology*.

[19] Hawco S., Rolnik D. L., Woolner A. (2022). The impact of mitigation measures on perinatal outcomes during the first nine months of the COVID-19 pandemic: a systematic review with meta-analysis. *European Journal of Obstetrics, Gynecology, and Reproductive Biology*.

[20] Pilarska I., Bizon M., Sawicki W. (2023). Influence of COVID-19 infection on placental function. *Ginekologia Polska*.

[21] Jeganathan K., Paul A. B. (2022). Vertical transmission of SARS-CoV-2: a systematic review. *Obstetric Medicine*.

[22] Meng X., Zhu K., Wang J., Liu P. (2022). Can SARS-CoV-2 positive pregnant women affect the hearing of their newborns: a systematic review. *American Journal of Otolaryngology*.

[23] Aljohani M. A., Albalawi F. M., Albalawi B. M. (2022). Consequences of SARS-CoV-2 infection in pregnant women and their infants: a systematic review. *Cureus*.

[24] Panda S. K., Mishra A., Pathak M. (2022). Clinical outcome of neonates born to SARS-CoV-2 positive mothers in India: a systematic review and meta-analysis. *Cureus*.

[25] Smith E. R., Oakley E., Grandner G. W. (2023). Adverse maternal, fetal, and newborn outcomes among pregnant women with SARS-CoV-2 infection: an individual participant data meta-analysis. *Health*.

[26] Sheikh J., Lawson H., Allotey J. (2022). Global variations in the burden of SARS-CoV-2 infection and its outcomes in pregnant women by geographical region and country's income status: a meta-analysis. *Health*.

[27] Deng J., Ma Y., Liu Q., Min D., Liu M., Liu J. (2022). Association of Infection with different SARS-CoV-2 variants during pregnancy with maternal and perinatal outcomes: a systematic review and meta-analysis. *International Journal of Environmental Research and Public Health*.

[28] Yang J., D’Souza R., Kharrat A. (2022). Coronavirus disease 2019 pandemic and pregnancy and neonatal outcomes in general population: a living systematic review and meta-analysis (updated Aug 14, 2021). *Acta Obstetricia et Gynecologica Scandinavica*.

[29] Aho Glele L. S., Simon E., Bouit C. (2022). Association between SARS-Cov-2 infection during pregnancy and adverse pregnancy outcomes: a re-analysis of the data reported by Wei et al. (2021). *Infectious Diseases Now*.

[30] Prasad S., Kalafat E., Blakeway H. (2022). Systematic review and meta-analysis of the effectiveness and perinatal outcomes of COVID-19 vaccination in pregnancy. *Nature Communications*.

[31] Rawal S., Tackett R. L., Stone R. H., Young H. N. (2022). COVID-19 vaccination among pregnant people in the United States: a systematic review. *American Journal of Obstetrics & Gynecology MFM*.

[32] Novillo B., Martínez-Varea A. (2023). COVID-19 vaccines during pregnancy and breastfeeding: a systematic review. *Journal of Personalized Medicine*.

[33] De Rose D. U., Salvatori G., Dotta A., Auriti C. (2022). SARS-CoV-2 vaccines during pregnancy and breastfeeding: a systematic review of maternal and neonatal outcomes. *Viruses*.

[34] Magnus M. C., Örtqvist A. K., Dahlqwist E. (2022). Association of SARS-CoV-2 vaccination during pregnancy with pregnancy outcomes. *Journal of the American Medical Association*.

[35] Krishna H., Motwani R., Kumari C. (2022). Evaluation of safety concerns for COVID-19 immunization of pregnant women: a systematic review of emerging evidence. *Maedica*.

[36] Shafiee A., Kohandel Gargari O., Teymouri Athar M. M., Fathi H., Ghaemi M., Mozhgani S. H. (2023). COVID-19 vaccination during pregnancy: a systematic review and meta-analysis. *BMC Pregnancy and Childbirth*.

[37] Hagrass A. I., Almadhoon H. W., Al-Kafarna M. (2022). Maternal and neonatal safety outcomes after SAR-CoV-2 vaccination during pregnancy: a systematic review and meta-analysis. *BMC Pregnancy and Childbirth*.

[38] Ding C., Liu Y., Pang W., Zhang D., Wang K., Chen Y. (2023). Associations of COVID-19 vaccination during pregnancy with adverse neonatal and maternal outcomes: a systematic review and meta-analysis. *Frontiers in Public Health*.

[39] Kontovazainitis C. G., Katsaras G. N., Gialamprinou D., Mitsiakos G. (2023). COVID-19 vaccination and pregnancy: a systematic review of maternal and neonatal outcomes. *Journal of Perinatal Medicine*.

[40] Burwick R. M., Dellapiana G., Newman R. A. (2022). Complement blockade with eculizumab for treatment of severe coronavirus disease 2019 in pregnancy: a case series. *American Journal of Reproductive Immunology*.

[41] Ferrugini C. L. P., Boldrini N. A. T., Costa F. L. S., Salgueiro M. A. O. B., Coelho P. D. P., Miranda A. E. (2022). SARS-CoV-2 infection in pregnant women assisted in a high-risk maternity hospital in Brazil: clinical aspects and obstetric outcomes. *PLoS One*.

[42] Provenzi L., Mambretti F., Villa M. (2021). Hidden pandemic: COVID-19-related stress, *SLC6A4* methylation, and infants’ temperament at 3 months. *Scientific Reports*.

[43] Colson A., Depoix C. L., Dessilly G. (2021). Clinical and *in vitro* evidence against placenta infection at term by severe acute respiratory syndrome coronavirus 2. *The American Journal of Pathology*.

[44] Cosma S., Carosso A. R., Corcione S. (2021). Longitudinal analysis of antibody response following SARS-CoV-2 infection in pregnancy: from the first trimester to delivery. *Journal of Reproductive Immunology*.

[45] Oncel M. Y., Akın I. M., Kanburoglu M. K. (2021). A multicenter study on epidemiological and clinical characteristics of 125 newborns born to women infected with COVID-19 by Turkish Neonatal Society. *European Journal of Pediatrics*.

[46] Khoury R., Bernstein P. S., Debolt C. (2020). Characteristics and outcomes of 241 births to women with severe acute respiratory syndrome coronavirus 2 (SARS-CoV-2) infection at five New York City Medical Centers. *Obstetrics & Gynecology*.

[47] Jamieson D. J., Rasmussen S. A. (2022). An update on COVID-19 and pregnancy. *American Journal of Obstetrics and Gynecology*.

[48] Carrasco I., Muñoz-Chapuli M., Vigil-Vázquez S. (2021). SARS-CoV-2 infection in pregnant women and newborns in a Spanish cohort (GESNEO-COVID) during the first wave. *BMC Pregnancy and Childbirth*.

[49] Knight M., Bunch K., Vousden N. (2020). Characteristics and outcomes of pregnant women admitted to hospital with confirmed SARS-CoV-2 infection in UK: national population based cohort study. *BMJ*.

[50] Wang C.-L., Liu Y.-Y., Wu C.-H., Wang C.-Y., Wang C.-H., Long C.-Y. (2021). Impact of COVID-19 on pregnancy. *International Journal of Medical Sciences*.

[51] Prochaska E., Jang M., Burd I. (2020). COVID-19 in pregnancy: placental and neonatal involvement. *American Journal of Reproductive Immunology*.

[52] Schwartz D. A. (2020). An analysis of 38 pregnant women with COVID-19, their newborn infants, and maternal-fetal transmission of SARS-CoV-2: maternal coronavirus infections and pregnancy outcomes. *Archives of Pathology & Laboratory Medicine*.

[53] Caparros-Gonzalez R. A. (2020). Consecuencias maternas y neonatales de la infección por coronavirus COVID-19 durante el embarazo: una scoping review. *Revista Española de Salud Pública*.

[54] Naidu S. A. G., Clemens R. A., Pressman P. (2022). COVID-19 during pregnancy and postpartum. *Journal of Dietary Supplements*.

[55] Kyle M. H., Glassman M. E., Khan A. (2020). A review of newborn outcomes during the COVID-19 pandemic. *Seminars in Perinatology*.

[56] Friedman D. M., Kim M., Costedoat-Chalumeau N. (2020). Electrocardiographic QT intervals in infants exposed to hydroxychloroquine throughout gestation. *Circulation. Arrhythmia and Electrophysiology*.

[57] Papapanou M., Papaioannou M., Petta A. (2021). Maternal and neonatal characteristics and outcomes of COVID-19 in pregnancy: an overview of systematic reviews. *International Journal of Environmental Research and Public Health*.

[58] Ayed A., Embaireeg A., Benawadh A. (2020). Maternal and perinatal characteristics and outcomes of pregnancies complicated with COVID-19 in Kuwait. *BMC Pregnancy and Childbirth*.

[59] Elsaddig M., Khalil A. (2021). Effects of the COVID pandemic on pregnancy outcomes. *Best Practice & Research. Clinical Obstetrics & Gynaecology*.

[60] Ko J. Y., DeSisto C. L., Simeone R. M. (2021). Adverse pregnancy outcomes, maternal complications, and severe illness among US delivery hospitalizations with and without a coronavirus disease 2019 (COVID-19) diagnosis. *Clinical Infectious Diseases*.

[61] Wastnedge E. A. N., Reynolds R. M., van Boeckel S. R. (2021). Pregnancy and COVID-19. *Physiological Reviews*.

[62] Jaiswal N., Puri M., Agarwal K. (2021). COVID-19 as an independent risk factor for subclinical placental dysfunction. *European Journal of Obstetrics, Gynecology, and Reproductive Biology*.

[63] Mirbeyk M., Saghazadeh A., Rezaei N. (2021). A systematic review of pregnant women with COVID-19 and their neonates. *Archives of Gynecology and Obstetrics*.

[64] Wainstock T., Yoles I., Sergienko R., Sheiner E. (2021). Prenatal maternal COVID-19 vaccination and pregnancy outcomes. *Vaccine*.

[65] Huang C., Wang Y., Li X. (2020). Clinical features of patients infected with 2019 novel coronavirus in Wuhan, China. *Lancet*.

[66] Sun P., Lu X., Xu C., Sun W., Pan B. (2020). Understanding of COVID-19 based on current evidence. *Journal of Medical Virology*.

[67] Karlberg J., Chong D. S. Y., Lai W. Y. Y. (2004). Do men have a higher case fatality rate of severe acute respiratory syndrome than women do?. *American Journal of Epidemiology*.

[68] Kalinka J., Wielgos M., Leszczynska-Gorzelak B. (2021). COVID-19 impact on perinatal care: risk factors, clinical manifestation and prophylaxis. Polish experts’ opinion for December 2020. *Ginekologia Polska*.

[69] Castro P., Matos A., Werner H., Lopes F. P., Tonni G., Araujo Júnior E. (2020). Covid-19 and pregnancy: an overview. *Revista Brasileira de Ginecologia e Obstetrícia/RBGO Gynecology and Obstetrics*.

[70] Edlow A. G., Li J. Z., Collier A. R. J. (2020). assessment of maternal and neonatal SARS-CoV-2 viral load, transplacental antibody transfer, and placental pathology in pregnancies during the COVID-19 pandemic. *JAMA Network Open*.

[71] Musa S. S., Bello U. M., Zhao S., Abdullahi Z. U., Lawan M. A., He D. (2021). Vertical transmission of SARS-CoV-2: a systematic review of systematic reviews. *Viruses*.

